# Recurrent Meniscal Instability Requiring Revision Repair 28 Years After the Initial Repair for a Bucket Handle Medial Meniscus Tear: A Case Report

**DOI:** 10.1155/cro/7395591

**Published:** 2025-08-29

**Authors:** Shintaro Onishi, Tomoya Iseki, Ryo Kanto, Hiroshi Nakayama, Motoi Yamaguchi, Toshiya Tachibana, Shinichi Yoshiya

**Affiliations:** ^1^Department of Orthopaedic Surgery, Nishinomiya Kaisei Hospital, Nishinomiya, Hyogo, Japan; ^2^Department of Orthopaedic Surgery, Hyogo Medical University, Nishinomiya, Hyogo, Japan; ^3^Department of Orthopaedic Surgery, Meiwa Hospital, Nishinomiya, Hyogo, Japan

**Keywords:** knee, long term, meniscal repair, osteoarthritis, revision meniscal repair

## Abstract

In recent decades, arthroscopic meniscal repair has been increasingly indicated for meniscal tears in the last decades. Although literature generally reports favorable surgical outcomes, it remains unclear whether the repaired meniscus maintains its function over the long term while performing its chondroprotective function without recurrent tear after clinical healing. A 43-year-old Japanese man who underwent meniscal repair for a bucket handle tear of the medial meniscus (MM) at the age of 15 years presented with right knee pain and catching symptoms without a preceding traumatic event. Physical examination revealed joint line tenderness and a catching sensation with rotating motion in the medial aspect of the knee. Plain radiographs showed no apparent osteoarthritic changes. Magnetic resonance imaging (MRI) showed infiltration of joint fluid at the interface between the MM and the joint capsule. Based on the patient's history and the examination results, a retear of the MM was diagnosed, and revision MM repair was conducted using the inside-out technique. At the 2-year follow-up, the patient remained asymptomatic, physical examination revealed no meniscal symptoms, and radiological examination showed no signs of osteoarthritic progression. The present study details a case involving a retear of a repaired meniscus without an inciting traumatic event after a 28-year asymptomatic period is reported. The long-term results of meniscus repair surgery performed more than 20 years ago have rarely been reported in the literature. This case report indicates that the long-term preservation of articular cartilage following successful meniscal repair can be attributed to the maintenance of meniscal function. On the other hand, even after clinical healing has been achieved, there is a possibility that the risk of meniscal retear will continue over time.

## 1. Introduction

Arthroscopic meniscectomy has been the most frequent surgical treatment for meniscal tears, and studies have shown favorable outcomes for partial meniscectomy performed on stable knees [1, 2]. However, meniscectomy has also been reported to cause highly prevalent posttraumatic osteoarthritic changes in the long term [3, 4]. Based on these previous findings, the importance of repairing the meniscus while preserving its function has been emphasized over the last decade. As a result, the number of meniscal repairs has increased, especially in younger, active patient populations. Among the various types of tears, longitudinal or bucket handle tears in vascular lesions are known to be a good indication, with many reports demonstrating favorable outcomes after arthroscopic meniscal repair [5–7]. However, the high failure rate of 20%–40% reported in long-term follow-up studies of arthroscopic meniscal repair raises concern [8–11]. Another drawback is that 14% of repaired menisci retear years after healing has been arthroscopically confirmed [12].

Although some studies have reported long-term results after meniscal repair, they have included only a few cases that were followed for more than 20 years [13, 14]. As a result, it remains unclear whether the repaired meniscus will maintain its physiological function long after it has healed. In addition, the optimal treatment option for retorn menisci after primary meniscal repair is also controversial [15–18].

This report describes a case of repeat meniscal tearing 28 years after surgical correction of a bucket handle medial meniscal (MM) tear. The initial repair yielded long-term symptomatic resolution and protected the articular cartilage until the retear, which occurred at the original repair site despite no evidence of a traumatic event. Revision repair was performed, and symptoms were alleviated for up to 2 years.

## 2. Case Presentation

**Figure 1:**
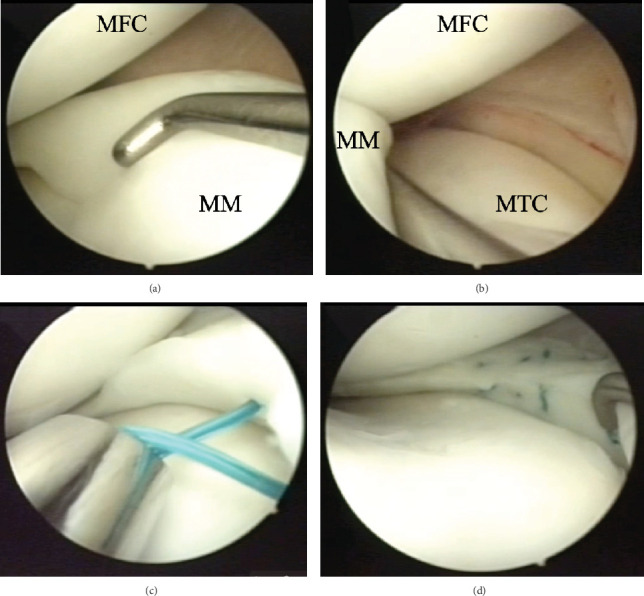
Arthroscopic images at the initial meniscal repair at the age of 15 years. (a, b) Bucket handle tear of the medial meniscus (MM) at the peripheral vascular zone from the anteromedial part to the posterior horn. (c, d) Meniscal repair using the inside-out technique was performed to secure the meniscal stability.

A 43-year-old Japanese man presented at our clinic complaining of right knee pain and catching symptoms without a history of a preceding traumatic event. Twenty-eight years before the visit (at the age of 15), he underwent a meniscal repair for a bucket handle tear of the MM occurring after sports-related injuries (Figure 1). The initial arthroscopic meniscal repair was performed using the inside-out technique with 2-0 nonabsorbable sutures for a traumatic bucket handle tear in the peripheral vascular area, which extended from the anteromedial part to the posterior horn (Video S1).

**Figure 2:**
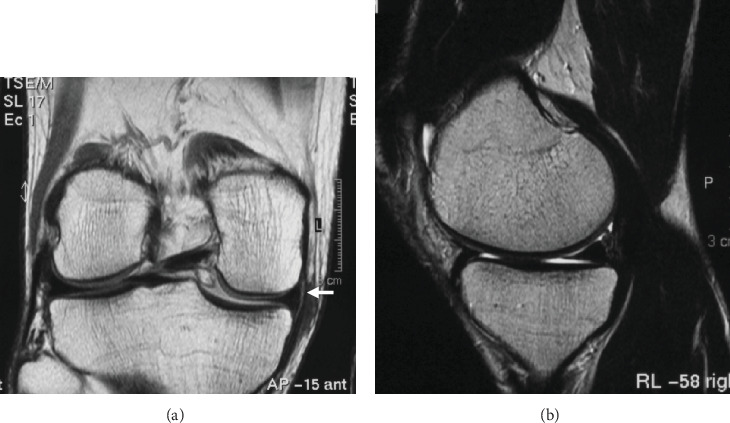
Magnetic resonance imaging findings taken at 8 years after the initial meniscal repair for the bucket handle tear of the medial meniscus. (a, b) The repaired site appears healed, and no intrameniscal degeneration is observed (white arrow).

The patient returned to sports and daily activity after the initial surgery. When he was 23, 8 years after the initial injury, the patient revisited the clinic complaining of mild pain and discomfort on the medial side of the knee. However, at the time, no symptoms such as catching, locking, or signs of meniscal distress were present. Magnetic resonance imaging (MRI) showed complete healing of the repaired meniscus without cartilage wear (Figure 2), and the symptoms dissolved after a short period of time. Pain and mechanical symptoms associated with the meniscus had been completely resolved for over 28 years, indicating that healing of the repaired site had been achieved and maintained. However, 28 years after the initial surgery, the patient visited our outpatient clinic because of recurrent medial knee pain unrelated to any recent trauma.

**Figure 3:**
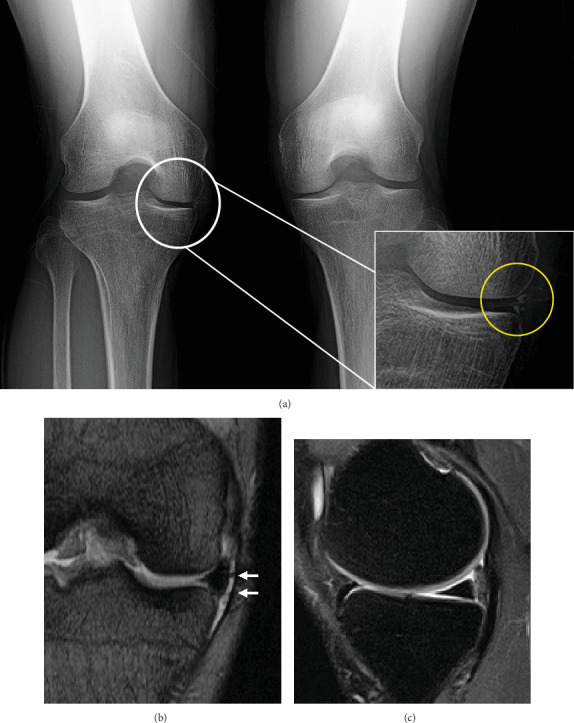
A 43-year-old male who underwent meniscal repair 28 years ago for a bucket handle tear of the MM in the right knee. (a) Preoperative plain radiograph using the Rosenberg view shows no significant osteoarthritic changes except for calcification of the medial joint space (yellow dotted circle). (b) MRI reveals infiltration of joint fluid at the interface between the MM and the joint capsule without cartilage damage. (c) In the sagittal view, no apparent tearing or degenerative changes in the posterior horn of the MM are observed. MM, medial meniscus.

Physical examination of the right knee revealed joint line tenderness and a catching sensation associated with the McMurray test maneuver in the medial aspect of the knee. The patient had full range of motion, and there were no signs indicative of ligamentous instability or increased general joint laxity. A plain radiograph with a Rosenberg view of the knee showed no osteoarthritic changes except for small, spotty calcifications in the medial joint space (Figure 3). A whole-leg radiograph showed a mild varus alignment corresponding to a hip-knee-ankle angle of 6.0° varus with a medial proximal tibial angle of 85.1° and a medial posterior tibial slope angle of 5.7°. MRI showed joint fluid infiltration at the interface between the MM and the joint capsule, though no obvious tearing or degenerative changes were observed within the meniscal body (Figure 3). The patient's history and physical examination results suggested that instability at the originally repaired site was causing the catching symptom. Therefore, a diagnosis of MM retear was made.

**Figure 4:**
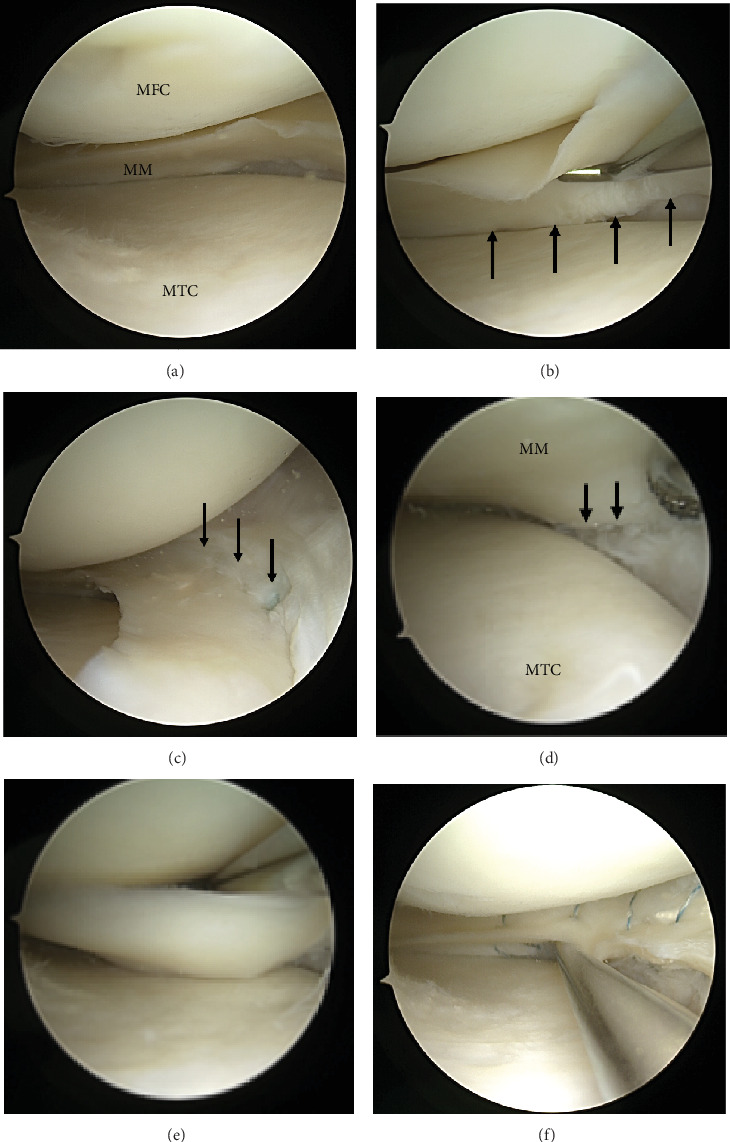
Arthroscopic findings in the setting of the revision meniscal repair 28 years after the initial meniscal repair. (a) There was minimal chondral damage in the medial compartment. (b–d) Unstable peripheral retear of the repaired meniscus with loosening at the initial repaired site of the meniscocapsular junction (black arrow). (e) Meticulous probing shows meniscal instability. (f) Stability of the meniscus was improved after revision meniscal repair using the inside-out technique. MFC, medial femoral condyle; MM, medial meniscus; MTC, medial tibial condyle.

Consequently, repeat arthroscopic MM repair using the inside-out technique was indicated and performed. During revision surgery, there was minimal to no chondral damage in the medial compartment (Figure 4). extending from the anteromedial part to the posterior horn (Figures 4, 4, 4, and 4). The tear site was stabilized via inside-out revision meniscal repair, combined with biological augmentation by rasping the tear site and a bone marrow venting procedure (Figure 4).

**Figure 5:**
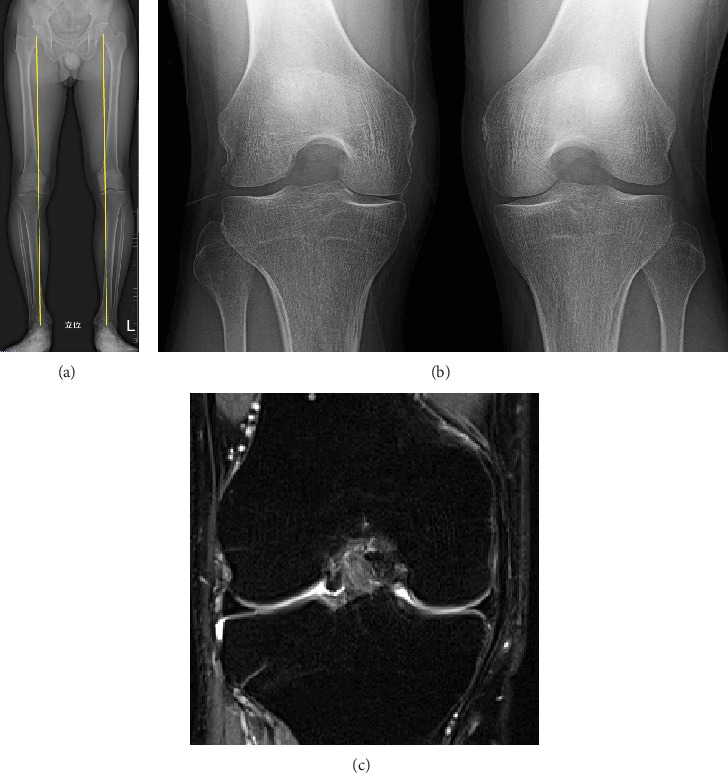
Postoperative images taken 2 years after revision meniscal repair. (a) The whole leg standing radiograph shows the same extent of degree of varus alignment in the bilateral leg. (b) Postoperative plain radiograph using the Rosenberg view shows no further progression of osteoarthritic changes in the right knee compared to the preoperative images. (c) MRI demonstrated continuity between the MM and the joint capsule without joint fluid infiltration at the interface.

At the 2-year follow-up after the revision surgery, the patient remained asymptomatic and physical examination revealed no mechanical symptoms. Plain radiographs showed no progression of osteoarthritic changes in the affected knee joint and MRI demonstrated continuity between the MM and the joint capsule without joint fluid infiltration at the interface (Figure 5). This report has been approved by the authors' affiliated institutions.

## 3. Discussion

Although a high success rate has been reported following arthroscopic meniscal repair performed for peripheral meniscal tears [6, 19, 20], long-term performance of the repaired meniscus has not been clarified. A recently published review paper on mid- to long-term follow-up studies of meniscal repair reported a revision surgery (failure) rate of 19% over an average follow-up period of 86 months [21]. Regarding the long-term results of studies with a follow-up period of more than 10 years, Hagmeijer conducted an 18-year follow-up study of patients aged 18 years and younger who underwent isolated meniscal repair surgery and reported an overall failure rate of 42% [9]. Another study by Noyes et al. reported an overall failure rate of 38% with a minimum 10-year follow-up [11]. As for the long-term radiological outcomes, Kalifis et al. reported that successful repairs were associated with less osteoarthritic progression at a median follow-up of 114 months and that the chondroprotective effect outweighs the high failure risk [22]. The present case demonstrated that the repaired meniscus had exhibited a chondroprotective effect and remained asymptomatic for over 28 years. Based on the experience of this case, meniscal repair should be considered for bucket handle tears, especially in the young population, as it is expected to preserve meniscal function in the long term.

Retear of the repaired meniscus after healing has not been well investigated in the literature. Kurosaka et al. identified retear of the repaired menisci in 13 of 90 cases (14.4%) after clinical healing was confirmed by second-look arthroscopy. Retear occurred, on average, 48 months after the initial repair and was associated with high postoperative levels of activity [12]. The authors have stated that inadequate strength of the reparative tissue at the repair site may be one of the factors related to the high failure rate in active patient populations. Previous MRI follow-up studies following meniscal repair have demonstrated that continued abnormal signal intensity persists at the repaired site in the majority of the clinically healed repaired menisci [23, 24]. Furthermore, animal studies in a canine model have shown a distinct difference in histology between the repaired tissue at the defect site and the surrounding native meniscal tissue even at 6 months after surgery [25, 26]. In the present case, the retear of the repaired meniscus occurred at the initial site of repair without a traumatic event, even after a significant amount of time had passed. The results of the previous relevant studies, as well as our experiences with this case, seem to indicate that the repaired tissue may not be able to regain the native meniscal properties and that continuous follow-up observation is necessary even after apparent clinical healing has been confirmed.

The optimal treatment option for retears following primary meniscal repair has not yet been clearly established. Krych et al. reported that the clinical failure occurred in 7 of 34 patients (21%) who underwent revision meniscal repair at a mean follow-up of 6 years [27]. Jackson et al. conducted a meta-analysis of the four previous studies dealing with revision meniscal repair and reported an overall failure rate of 25.3%, with a mean follow-up of 4.9 years [17]. The success rates reported in these previous studies are comparable or slightly inferior to the results of previous studies on primary meniscal repair [5, 24]. In the present case, revision repair with biological augmentation was performed, resulting in satisfactory clinical and radiological results at 2 years; however, further follow-up is needed to clarify the significance of revision meniscal repair.

A case of retear of the repaired meniscus without an inciting traumatic event after a 28-year asymptomatic period is reported. To the best of our knowledge, this is a case with the longest follow-up ever reported in literature with images and video at initial surgery available. In conclusion, this case report indicates long-term preservation of articular cartilage following successful meniscal repair, which is attributed to the preservation of meniscal function, even though a high revision surgery rate was reported in the literature. On the other hand, the risk of meniscal retear may persist for many years even after clinical healing has been achieved. This information may provide valuable insights into the long-term function of the repaired menisci and may be useful in the management of patients who experience retearing at the repair site.

## Data Availability

The data that support the findings of this study are available from the corresponding author upon reasonable request.

## Supporting Information

Supporting Information Video S1. Arthroscopic video taken during initial meniscal repair for a bucket handle tear of the medial meniscus.
